# Coordination abilities and movement organization during the fencing lunge in youth foil fencers: an exploratory biomechanical study

**DOI:** 10.3389/fspor.2026.1934907

**Published:** 2026-09-17

**Authors:** Lina Hussein, Hasan Ashkanani, Laila Al Kilani, Samer AbuEid, Hatem Shlool, Hashem Kilani

**Affiliations:** 1 Department of Physical Education, Jerash University, Jerash, Jordan; 2 College of Basic Education, The Public Authority for Applied Education and Training, Kuwait City, Kuwait; 3 Department of Physical Sport Sciences, Princess Nourah bint Abdulrahman University, Riyadh, Saudi Arabia; 4 Sport Sciences Department, Arab American University, Jenin, Palestine; 5 Physical and Health Education Department, Al-Ahliyya Amman University, Amman, Jordan; 6 Kinesiology and Exercise Sciences Department, The University of Jordan, Amman, Jordan

**Keywords:** biomechanics, coordination abilities, ecological dynamics, fencing, long-term athlete development, motor control, movement organization

## Abstract

**Background:**

Coordination abilities are considered fundamental determinants of motor performance and long-term athlete development, yet their relationship with the biomechanical characteristics of fencing performance remains poorly understood. Understanding these relationships may provide valuable insights for evidence-based coaching and athlete development.

**Objective:**

This exploratory study examined the associations between selected coordination abilities and kinematic variables of the fencing lunge in youth foil fencers.

**Methods:**

Nine competitive youth foil fencers (age: 11–15 years) completed a battery of seven coordination ability tests assessing spatial orientation, movement coupling, balance, rhythmic ability, adaptation ability, force differentiation, and reaction ability. Kinematic variables, including successful and unsuccessful defensive movement time, arm velocity, riposte velocity, and front-leg velocity, were obtained from two-dimensional video recordings using Kinovea motion analysis software. Spearman's rank-order correlation coefficients were calculated to examine associations between coordination abilities and biomechanical variables. Holm–Bonferroni correction was applied to account for multiple comparisons.

**Results:**

Before adjustment for multiple comparisons, very large positive correlations were observed between reaction ability and arm velocity during unsuccessful defensive actions (*ρ* = 0.833, *p* = 0.005) and between reaction ability and front-leg velocity during the fencing lunge (*ρ* = 0.817, *p* = 0.007). Correlations of *ρ* = 0.628 and *ρ* = −0.622 were also observed for adaptation ability with successful defensive movement time and movement coupling ability with unsuccessful defensive movement time, respectively. However, none of the associations remained statistically significant following Holm–Bonferroni correction. The coefficients are therefore reported descriptively and treated as candidate hypotheses for independent replication.

**Conclusion:**

After correction for multiple comparisons, this exploratory study found no statistically significant associations between the assessed coordination abilities and fencing-lunge kinematics. The observed coefficients do not provide confirmatory evidence that specific coordination abilities contribute to lunge performance; they identify candidate associations for future adequately powered studies. Future research should use larger samples and three-dimensional motion analysis.

## 1 Introduction

Fencing is a dynamic Olympic combat sport characterized by rapid offensive and defensive actions that require the precise integration of perceptual, cognitive, neuromuscular, and biomechanical processes (1–3). Successful performance depends on an athlete's ability to rapidly perceive an opponent's movements, process sensory information, make accurate tactical decisions, and execute technically efficient motor responses within fractions of a second (2, 4). Among the three Olympic fencing disciplines, foil places particularly high demands on movement precision, reaction speed, temporal coordination, and effective control of whole-body movement during offensive and defensive exchanges (1, 3). Consequently, competitive success in foil fencing depends not only on technical proficiency but also on the efficient interaction between neuromotor coordination and biomechanical movement execution (2–4).

Coordination abilities are fundamental determinants of motor performance and represent the foundation upon which complex sport-specific skills are acquired, refined, and executed efficiently (5–7). They enable athletes to regulate movement through the effective integration of sensory information, central nervous system processing, and neuromuscular control, thereby improving movement precision, timing, balance, and adaptability under constantly changing environmental conditions (6, 8). According to the theory of coordinative abilities proposed by Hirtz and further developed within motor learning and sport science, specific coordination abilities—including spatial orientation, movement coupling, balance, rhythmic ability, reaction ability, force differentiation, and adaptation to changing situations—constitute distinct yet interrelated components of athletic performance (5, 7, 9). During childhood and early adolescence, these abilities undergo rapid development and are considered highly trainable, making this developmental period particularly important for long-term athlete development and the acquisition of complex technical skills (8–10).

Although the classical theory of coordinative abilities provides a valuable framework for understanding motor performance, contemporary theories of motor control emphasize that skilled movement emerges through the continuous interaction between the athlete, the task, and the surrounding environment (11–13). Within the ecological dynamics framework, movement is viewed as an adaptive process in which athletes continuously perceive environmental information and reorganize their actions to satisfy changing task constraints (11, 12). Rather than relying solely on pre-programmed motor patterns, expert performers develop flexible movement solutions that enable them to respond rapidly and efficiently to unpredictable competitive situations (13, 14). This perspective is particularly relevant in fencing, where athletes must constantly integrate visual information, anticipate an opponent's intentions, and execute technically precise movements within extremely short time intervals (15, 16). Nonlinear pedagogy and constraints-led approaches further emphasize adaptable movement solutions in representative tasks (17, 18). Ecological accounts of sport decision making and movement variability likewise describe actions as emerging under changing constraints (19, 20).

From a biomechanical perspective, the fencing lunge is the fundamental offensive action in foil fencing because it enables athletes to reduce the distance to the opponent while maintaining weapon accuracy and dynamic postural stability (4, 21, 22). Successful execution depends on coordinated lower- and upper-limb sequencing, rapid force generation, and precise control of weapon trajectory (4, 5, 22). Ecological dynamics interprets skilled movement as an adaptive response to interacting individual, task, and environmental constraints (6, 11–13). In fencing, this adaptation requires continuous perception–action coupling as the athlete responds to an opponent’s movements in real time (15, 19). Biomechanical studies indicate that target distance, lower-limb kinematics, and intersegmental coordination influence lunge execution (4, 5, 22). Integrating coordination assessment with biomechanical analysis may therefore offer a broader description of fencing performance than examining isolated kinematic variables.

Despite increasing interest in fencing biomechanics, previous investigations have largely examined kinematic or physiological characteristics rather than the relationship between multiple coordination abilities and lunge organization, particularly in youth athletes (2–5, 22). Much of the available biomechanical evidence concerns adult or higher-level athletes, leaving limited evidence on coordination during early adolescence (3, 22). Coaches therefore have little empirical guidance on which coordination abilities, if any, relate to fencing-specific movement organization during long-term athlete development (9, 10).

Therefore, the purpose of this exploratory study was to investigate the associations between selected coordination abilities and the kinematic characteristics of the fencing lunge in youth foil fencers aged 11–15 years. Specifically, the study examined the relationships between seven coordination abilities—spatial orientation, movement coupling, balance, rhythmic ability, adaptation ability, force differentiation, and reaction ability—and six biomechanical variables describing movement time and segmental velocity during fencing performance. It was hypothesized that athletes demonstrating superior coordination abilities would exhibit more favorable lunge kinematics. Given the exploratory design and limited sample size, the findings are intended to provide preliminary evidence to guide future biomechanical investigations and evidence-based training programmes for developing fencers. Accordingly, the study should be regarded as hypothesis-generating rather than hypothesis-confirming.

## 2 Methods

### 2.1 Study design

This exploratory cross-sectional study investigated the associations between selected coordination abilities and the kinematic characteristics of the fencing lunge in youth foil fencers. The study was designed to examine whether individual differences in coordination abilities were associated with biomechanical variables describing defensive movement time and segmental movement velocity during the fencing lunge. Because the primary objective was to explore relationships rather than establish causal effects, an observational analytical design was adopted. Ethical approval for the study was obtained from the Institutional Review Board (IRB) of The University of Jordan (Decision No. 474/2025). All participants and their parents or legal guardians provided written informed consent before testing commenced.

### 2.2 Participants

Nine youth foil fencers (six males and three females) voluntarily participated in this study. Participants ranged in age from 11 to 15 years and were registered competitive fencers who trained regularly under the supervision of qualified fencing coaches. At the time of testing, all participants were free from musculoskeletal injury or any medical condition that could affect fencing performance or participation in physical activity.

Participant characteristics are presented in Table 1. The mean (± SD) age was 13.67 ± 1.41 years, height 1.65 ± 0.11 m, body mass 56.83 ± 11.42 kg, and fencing experience 2.38 ± 1.60 years.

**Table 1:** Participant characteristics of the youth foil fencers (*n* = 9).

Variable	Mea*n* ± SD	Minimum	Maximum
Age (years)	13.67 ± 1.41	11.0	15.0
Height (m)	1.65 ± 0.11	1.47	1.79
Body mass (kg)	56.83 ± 11.42	40.7	72.3
Training experience (years)	2.38 ± 1.60	1.0	6.0

Sex distribution.

Male: 6 (66.7%).

Female: 3 (33.3%).

Because youth foil fencers within this age category represent a limited competitive population, all eligible athletes meeting the inclusion criteria were recruited, resulting in a census sample rather than a randomly selected sample.

### 2.3 Assessment of coordination abilities

Coordination abilities were evaluated using seven standardized field-based motor tests selected according to the theoretical framework of coordinative abilities proposed by Hirtz and by Meinel and Schnabel, together with previous studies involving youth athletes and fencing performance. Test selection was based on evidence from the literature and expert evaluation to ensure that the assessment battery adequately represented the principal components of coordinative ability relevant to fencing performance.

Prior to testing, all participants completed a standardized 15-minute warm-up consisting of light running, dynamic stretching, and fencing-specific movement drills. Each participant then received verbal instructions and a demonstration of every test, followed by one familiarization trial before formal testing commenced. All assessments were administered by the same investigator under identical environmental conditions to minimize measurement variability. When more than one trial was permitted, the best performance was retained for subsequent statistical analysis.

The seven coordination abilities assessed, the corresponding field tests, and measurement units are summarized in Table 2.

**Table 2:** Coordination abilities, assessment tests, outcome measures and performance criteria.

Coordination ability	Assessment test	Outcome measure	Performance criterion
Spatial orientation	Forward–backward jump test (15 s)	Number of successful jumps	Higher score=better performance
Movement coupling	Agility ladder with simultaneous ball passing	Completion time (s)	Lower time=better performance
Balance	One-leg stance test	Time maintained (s)	Longer time=better performance
Rhythmic ability	Side-to-side agility ladder test	Completion time (s)	Lower time=better performance
Adaptation ability	4 × 10 m shuttle run	Completion time (s)	Lower time=better performance
Force differentiation	Standing forward jump toward a 2.5 m target	Absolute error (cm)	Lower error=better performance
Reaction ability	V-test	Completion time (s)	Lower time=better performance

The assessment battery was developed based on the coordinative ability framework described by Hirtz and by Meinel and Schnabel and adapted from previously published protocols in fencing and youth motor performance research.

The coordination test battery was selected following a review of previous studies in motor coordination and fencing and was evaluated by a panel of specialists in biomechanics, motor learning, and fencing coaching to establish content validity before data collection.

### 2.4 Biomechanical data collection

Biomechanical testing was conducted at the Jordan Fencing Federation under standardized laboratory-like conditions. Each participant performed defensive fencing actions followed immediately by a direct riposte against an experienced coach acting as the attacking opponent.

Two synchronized Samsung NX30 digital video cameras (Samsung Electronics, South Korea) operating at 50 frames·s⁻¹ were used to record each trial. The sagittal-plane camera was positioned 5.50 m perpendicular to the direction of movement to capture the kinematics of the defensive action and riposte, whereas the frontal-plane camera was positioned 4.80 m in front of the testing area to verify the type and outcome of each defensive action. A one-meter calibration frame was positioned within the recording volume before data collection to permit spatial calibration during video analysis. Camera setup, spatial calibration, and displacement-based velocity calculations followed established biomechanical principles (23, 24). Video recordings were acquired at 50 Hz, providing a temporal resolution of 0.02 s per frame. This sampling frequency was used to identify movement onset and completion and to calculate event durations and displacement-based average velocities; it was not used to estimate instantaneous peak velocity or acceleration.

Reflective markers were attached to anatomical landmarks of the dominant upper and lower limbs, including the elbow, wrist, dorsum of the hand, knee, ankle, forefoot, and the weapon, to facilitate motion tracking during subsequent kinematic analysis. Marker placement was performed by the same investigator for all participants to ensure consistency across trials.

### 2.5 Experimental protocol

Each participant performed four standardized defensive trials followed immediately by a direct riposte. The attacking coach delivered randomized attacks directed toward the septime and octave target areas while the defending athlete responded using either a semicircular or diagonal parry according to the experimental protocol. To minimize anticipation effects, the sequence of attacks was randomized and was unknown to the participants before each trial.

The experimental protocol was designed to produce two successful and two unsuccessful defensive actions for each participant. A successful defensive action was operationally defined as a parry that intercepted and redirected the attacking blade, preventing the attack from reaching the valid foil target area before the direct riposte. An unsuccessful defensive action was defined as a failure to intercept or sufficiently redirect the attacking blade, allowing the attack to reach the valid target area. Each trial was classified by frame-by-frame review of the frontal-plane recording using these predefined outcome criteria. Classification was limited to whether the defensive action prevented a valid-target outcome; it did not involve adjudicating right-of-way or awarding the subsequent riposte under the full bout-refereeing rules.

For condition-specific outcomes, the two successful trials and two unsuccessful trials were averaged separately for each participant. Successful defensive movement time and successful-defense arm velocity were calculated from the two successful trials, whereas unsuccessful defensive movement time and unsuccessful-defense arm velocity were calculated from the two unsuccessful trials. Riposte velocity following successful defense was averaged across the two successful trials. Front-leg velocity was averaged across the four trials because a lunge was performed after each defensive action. These participant-level means were used in the correlation analyses.

### 2.6 Kinematic variables

Frame-by-frame digitization and event identification followed standard biomechanical and performance-analysis procedures (25, 26). Kinematic analysis was performed using Kinovea motion-analysis software (version 0.9.5, Kinovea Open Source Project), whose validity and reliability for two-dimensional measurements have been reported previously (27). Video recordings were calibrated before digitization using the one-meter reference frame positioned within the recording area. All digitization procedures were performed by the same investigator to minimize measurement error. Unless otherwise specified, the reported velocity outcomes represent average two-dimensional velocities calculated from displacement over elapsed time.

The following biomechanical variables were extracted from each trial:

Defensive movement time (successful defense)
Defensive movement time (unsuccessful defense)
Arm velocity during successful defense
Arm velocity during unsuccessful defense
Arm velocity during the direct riposte following successful defense
Front-leg velocity during the lunge

Front-leg velocity during the fencing lunge was determined using two-dimensional motion analysis in Kinovea. The displacement of the front foot marker between the initiation of the lunge and the instant of maximum extension was used to calculate average velocity (Figure 1).

**Figure 1:**
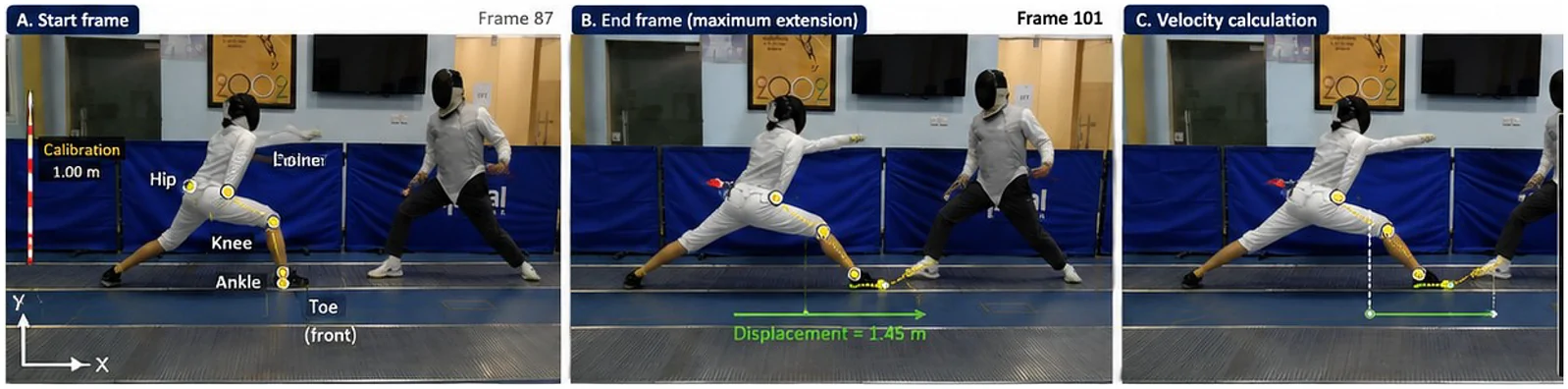
Measurement of front-leg velocity during the fencing lunge using Kinovea software.

The left panel illustrates the initial lunge posture as a simplified stick figure. The center panel presents the original Kinovea frame with reflective markers and trajectory analysis. The right panel illustrates the final posture at maximum lunge extension. The lower panel summarizes the measured variable, where front-leg velocity was calculated from the horizontal displacement of the toe marker (Δx) divided by the elapsed time (Δt) following spatial calibration of the recording area.

Arm velocity during the riposte was quantified by tracking the displacement of the weapon tip between the initiation and completion of the attacking movement using Kinovea software (Figure 2).

**Figure 2:**
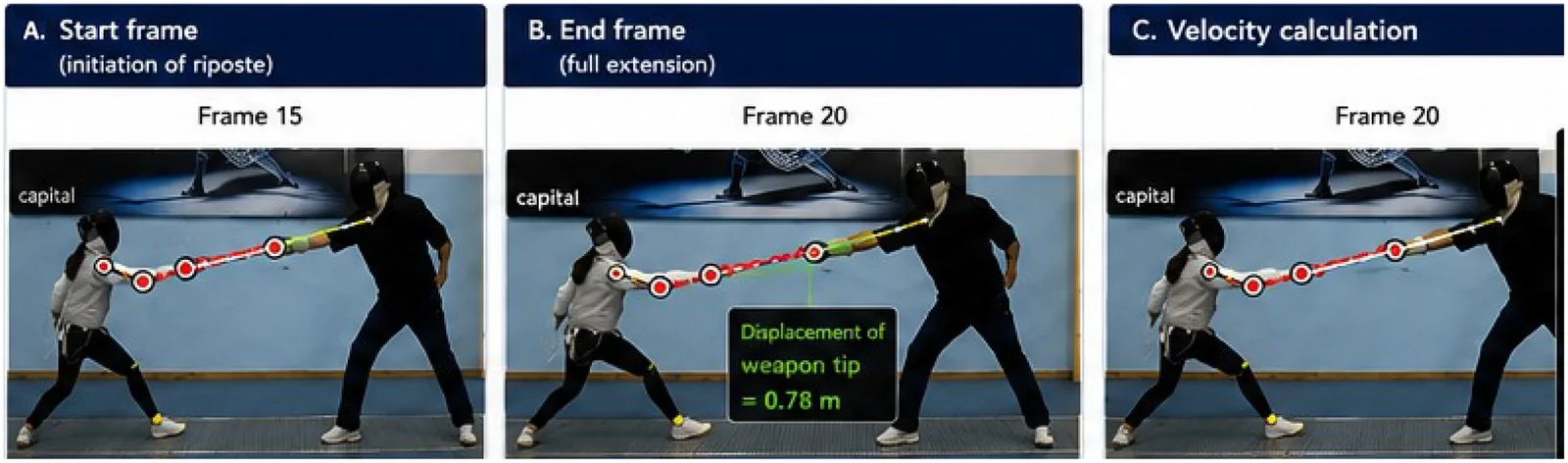
Measurement of arm (riposte) velocity using Kinovea software.

The left panel illustrates the initiation of the riposte as a stick figure. The center panel shows the original Kinovea frame with reflective markers and the tracked weapon-tip trajectory. The right panel illustrates the completion of the riposte. The lower panel summarizes the measured variable, where weapon-tip velocity was calculated from the displacement of the weapon marker (*Δ*x) divided by the movement time (*Δ*t).

Defensive movement time was defined as the interval between the initiation of the opponent's attack and the successful completion of the defensive action, as illustrated in Figure 3.

**Figure 3:**
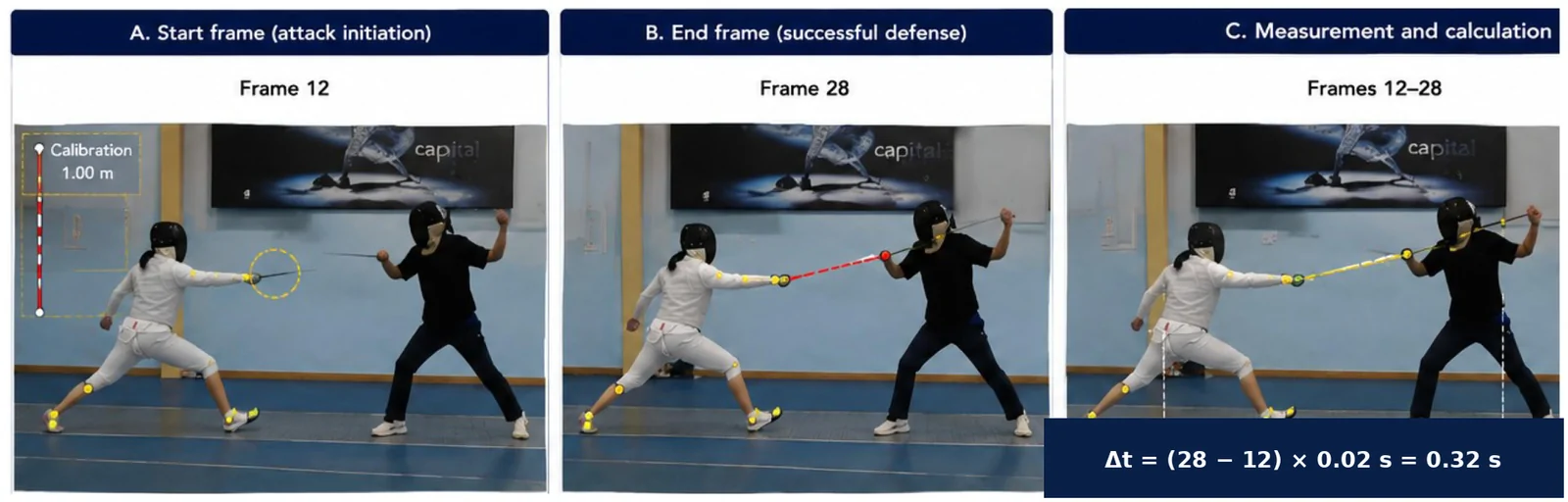
Measurement of defensive movement time during the fencing task using Kinovea software.

Panel A shows the initiation of the opponent's attack at Frame 12. Panel B shows the successful completion of the defensive action at Frame 28. Panel C shows the calculation of defensive movement time across Frames 12–28. At a recording frequency of 50 Hz (0.02 s per frame), defensive movement time was calculated as *Δ*t = (28 – 12) × 0.02 s = 0.32 s.

Defensive velocity was quantified by tracking the displacement of the weapon tip throughout the defensive movement using Kinovea software (Figure 4).

**Figure 4:**
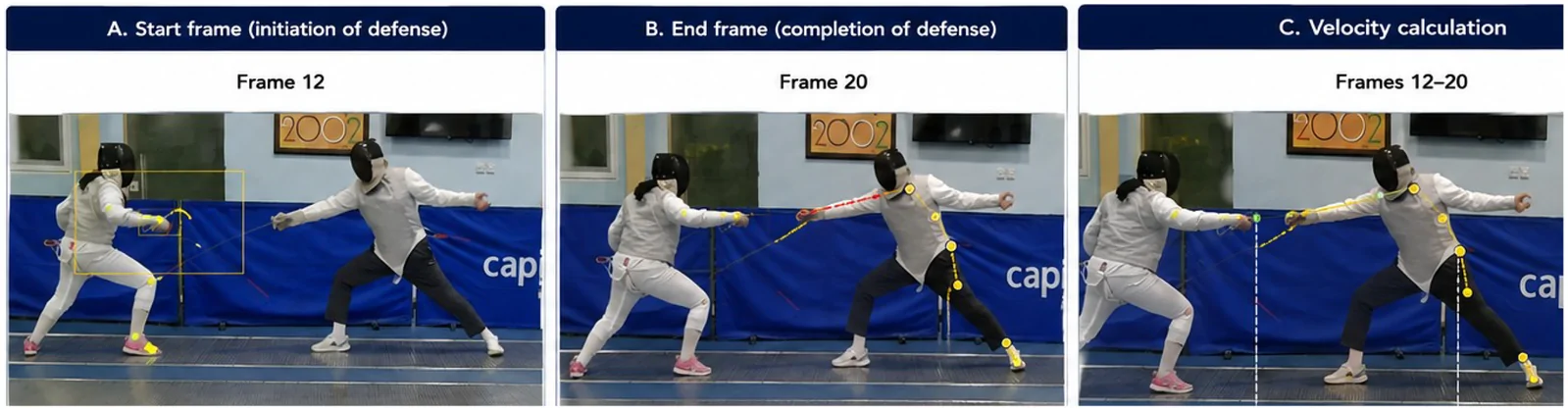
Measurement of defensive velocity using Kinovea software.

The left panel illustrates the initiation of the defensive movement as a stick figure. The center panel presents the original Kinovea frame with reflective markers and the tracked trajectory of the weapon tip during the defensive action. The right panel illustrates the completion of the defensive movement. The lower panel summarizes the measured variable, where defensive velocity was calculated from the displacement of the weapon tip (*Δ*x) divided by the elapsed movement time (*Δ*t).

These condition-specific participant-level means were used for all subsequent statistical analyses.

### 2.7 Statistical analysis

Descriptive statistics are presented as means ± standard deviations (SD), together with minimum and maximum values where appropriate. Data normality was evaluated using the Shapiro–Wilk test. Because several variables were not normally distributed and the sample size was small (*n* = 9), associations between coordination abilities and kinematic variables were examined using Spearman's rank-order correlation coefficients (*ρ*). To control the family-wise error rate resulting from multiple comparisons, Holm–Bonferroni adjusted *p*-values were calculated. Statistical significance was accepted at *p* < 0.05. All statistical analyses were performed using IBM SPSS Statistics version 26.0 (IBM Corp., Armonk, NY, USA).

#### 2.7.1 *Post hoc* power analysis

Given the small sample size (*n* = 9), a *post-hoc* power analysis was conducted to characterize the statistical power associated with the observed correlation effect sizes. The analysis was based on a two-tailed significance level of *α* = 0.05 and the observed Spearman correlation coefficients. For the largest observed association (*ρ* = 0.833), the estimated statistical power was approximately 0.93. However, power was substantially lower for moderate-sized associations; for example, an effect size of approximately *ρ* = 0.50 would provide only approximately 0.26 power with nine participants. These estimates indicate that the study had adequate sensitivity to detect very large associations but limited ability to detect moderate or smaller associations. Accordingly, nonsignificant findings should be interpreted cautiously, and the present results should be regarded as exploratory and hypothesis-generating rather than definitive.

Power was estimated using the noncentral t approximation for a two-tailed correlation test. Under this model, approximate achieved power was 0.89 for |*ρ*| = 0.817, 0.45 for |*ρ*| = 0.628, and 0.44 for |*ρ*| = 0.622. These values are descriptive, do not account fully for the stepwise Holm–Bonferroni procedure, and should not be interpreted as additional evidence for an association.

## 3 Results

Descriptive statistics were first calculated for participant characteristics, coordination abilities, and kinematic variables. Subsequently, Spearman's rank-order correlation coefficients were computed to examine associations between coordination abilities and lunge kinematics. To account for multiple testing, Holm–Bonferroni adjusted *p*-values were calculated.

### 3.1 Participant characteristics

The participants had a mean age of 13.67 ± 1.41 years, a mean height of 1.65 ± 0.11 m, a mean body mass of 56.83 ± 11.42 kg, and an average fencing experience of 2.38 ± 1.60 years (Table 1).

### 3.2 Descriptive statistics of coordination abilities

Descriptive statistics for the seven coordination abilities are presented in Table 3. Spatial Orientation Ability demonstrated the highest mean score (41.56 ± 5.98), whereas Reaction Ability showed the lowest completion time (2.06 ± 0.42 s). Variability among participants was generally low to moderate across the coordination assessments.

**Table 3:** Descriptive statistics of coordination abilities (*n* = 9).

Coordination ability	Mean ± SD	Minimum	Maximum
Spatial Orientation Ability	41.56 ± 5.98	27.00	47.00
Movement Coupling Ability	7.82 ± 1.39	6.38	10.00
Balance Ability (s)	40.78 ± 9.28	18.00	45.00
Rhythmic Ability	3.93 ± 1.23	2.84	7.00
Adaptation Ability	11.29 ± 1.40	10.06	14.56
Force Differentiation Ability	2.35 ± 1.38	1.29	5.90
Reaction Ability (s)	2.06 ± 0.42	1.43	2.97

### 3.3 Descriptive statistics of kinematic variables

Descriptive statistics for the kinematic variables measured during the fencing lunge are presented in Table 4. Mean defensive movement times ranged from 0.31 to 0.33 s, whereas mean arm velocity during the riposte reached 3.88 ± 0.73 m/s. Front-leg velocity during the lunge averaged 3.40 ± 0.72 m/s.

**Table 4:** Descriptive statistics of kinematic variables (*n* = 9).

Kinematic variable	Mean ± SD	Minimum	Maximum
Defensive movement time (successful defensive action)	0.31 ± 0.07 s	0.22	0.41
Defensive movement time (unsuccessful defensive action)	0.33 ± 0.10 s	0.18	0.48
Arm velocity during successful defense (m/s)	2.04 ± 0.36	1.28	2.54
Arm velocity during unsuccessful defense (m/s)	2.16 ± 0.93	1.23	4.18
Arm velocity during riposte (m/s)	3.88 ± 0.73	2.73	4.98
Front-leg velocity during the lunge (m/s)	3.40 ± 0.72	2.51	4.43

### 3.4 Associations between coordination abilities and lunge kinematics

Spearman's rank-order correlation analysis was performed to examine the associations between the seven coordination abilities and the six kinematic variables of the fencing lunge (Table 5). Complete coefficients, raw *p*-values, and Holm–Bonferroni adjusted *p*-values for all 42 comparisons are presented in Supplementary Table S1. Two coefficients were nominally significant before correction: Reaction Ability with arm velocity during unsuccessful defensive actions (*ρ* = 0.833, raw *p* = 0.005) and with front-leg velocity during the lunge (*ρ* = 0.817, raw *p* = 0.007). After Holm–Bonferroni correction, the adjusted *p*-values were 0.221 and 0.296, respectively; neither association remained statistically significant. The coefficients for Adaptation Ability with successful defensive movement time (*ρ* = 0.628, raw *p* = 0.070, adjusted *p* = 1.000) and Movement Coupling Ability with unsuccessful defensive movement time (*ρ* = −0.622, raw *p* = 0.074, adjusted *p* = 1.000) were also non-significant. Accordingly, all coefficients are reported descriptively and treated as candidate hypotheses rather than evidence of confirmed biomechanical relationships.

**Table 5:** Spearman’s rank-order correlations (*ρ*) between coordination abilities and kinematic variables of the fencing lunge (*n* = 9).

Coordination ability	Defensive time (successful)	Defensive time (unsuccessful)	Arm velocity (successful)	Arm velocity (unsuccessful)	Arm velocity (riposte)	Front-leg velocity
Spatial orientation (Change of position)	0.273	0.291	−0.033	−0.452	0.343	−0.494
Movement coupling	−0.042	−0.622	0.033	0.417	−0.250	0.367
Balance	0.298	0.311	−0.068	−0.183	0.525	−0.091
Rhythmic ability	0.059	−0.546	0.367	0.417	−0.067	0.567
Adaptation ability	0.628	0.134	−0.033	0.267	−0.400	0.417
Force differentiation	0.168	0.004	0.117	0.452	−0.243	0.318
Reaction ability	−0.025	−0.513	0.533	0.833*	0.183	0.817*

* Raw *p* < 0.05 before multiple-comparison correction.

Note. Values are Spearman’s rank-order correlation coefficients (*ρ*). * indicates nominal statistical significance before Holm–Bonferroni correction (raw *p* < 0.05). Complete correlation coefficients together with raw and Holm-adjusted *p*-values are presented in Supplementary Table S1.

Spearman's rank-order correlation coefficient (*ρ*) also provides an estimate of effect magnitude. These magnitudes are reported to describe patterns in the data, but the small sample and absence of statistical significance after Holm–Bonferroni correction preclude confirmatory inferences. The observed coefficients should not be characterized as meaningful relationships solely on the basis of their magnitude; instead, they identify candidate hypotheses for independent replication.

## 4 Discussion

### 4.1 Principal findings

The present exploratory study examined relationships between selected coordination abilities and the kinematic characteristics of the fencing lunge in youth foil fencers. After Holm–Bonferroni correction, no association met the adjusted significance threshold. The two largest coefficients involved Reaction Ability with arm velocity during unsuccessful defensive actions and with front-leg velocity, while Adaptation Ability and Movement Coupling Ability produced other comparatively large coefficients with defensive movement time. These estimates are descriptive signals only and do not establish associations in this sample. They are best treated as candidate hypotheses for future adequately powered studies. More generally, fencing performance requires coordinated perceptual, neuromuscular, and biomechanical processes (1, 2, 22); this provides a conceptual context for future testing rather than a conclusion established by the present inferential results.

### 4.2 Reaction ability and movement organization

Reaction Ability produced the two largest coefficients in the dataset. However, the corresponding Holm–Bonferroni adjusted *p*-values were 0.221 and 0.296, so neither relationship was statistically significant after correction. These observations therefore cannot demonstrate that reaction ability influences lunge kinematics; they identify candidate associations for replication. Independently of the present findings, foil fencing requires athletes to perceive an attack, select a response, and coordinate a parry and riposte within a short interval (1, 2). Ecological dynamics describes this type of behaviour in terms of perception–action coupling under changing constraints (6, 19), but the present study did not directly test that mechanism.

Ecological dynamics provides a theoretical framework for considering how skilled actions emerge from interactions among the athlete, task, and environment (6, 11, 19). The fencing lunge can be considered within this framework because its execution involves lower-limb propulsion, postural control, upper-limb coordination, and weapon trajectory (4, 5, 22). However, the present cross-sectional correlations do not determine whether athletes with better reaction ability organize these components more efficiently. Testing that proposition will require larger samples and longitudinal or experimental designs.

### 4.3 Adaptation ability and movement coupling

Adaptation Ability and Movement Coupling Ability produced comparatively large coefficients with defensive movement time, but neither coefficient was statistically significant before or after adjustment (raw *p* = 0.070 and 0.074; adjusted *p* = 1.000 for both). These estimates should not be interpreted as evidence of an association; they identify questions for future research. Conceptually, adaptation ability concerns modification of movement in response to changing demands, whereas movement coupling concerns the temporal and spatial coordination of body segments during complex actions (11–13).

Previous biomechanical studies indicate that lunge execution depends on coordinated lower-limb propulsion, trunk motion, and upper-limb movement (4, 5, 22). Changes in target distance and joint coordination can alter lunge kinematics (4, 5). Whether field-based coordination abilities are related to these kinematic patterns in youth fencers remains unresolved by the present dataset and requires confirmation in larger samples.

### 4.4 Implications for youth athlete development and coaching practice

Because no association remained statistically significant after correction, the present results do not provide an empirical basis for prescribing coordination training or identifying coordination measures as validated performance indicators. Reaction, adaptation, and movement-coupling tasks remain plausible candidates for future intervention research. Evidence-informed implementation also depends on coaches’ biomechanics literacy (28). Motor competence contributes to positive developmental trajectories, and long-term athlete development models emphasize progressive, age-appropriate capability development (29, 30). Accordingly, any fencing-specific application during early adolescence should be evaluated prospectively.

Long-term athlete development frameworks emphasize coordination, movement quality, and sport-specific skill acquisition during adolescence (9, 10). In this context, coaches may use representative tasks that challenge perceptual and motor processes, but this recommendation derives from established skill-acquisition principles rather than statistical confirmation from the present study (6, 15, 31). Controlled training studies are needed to determine whether such practice changes fencing biomechanics or competitive performance.

Representative practice can expose athletes to changing task constraints and require continuous adjustment of movement (6, 15, 31). Two examples warrant future evaluation. In a randomized choice-reaction parry–riposte drill, a coach delivers unpredictable attacks toward the septime or octave target area, and the athlete selects the appropriate parry before an immediate direct riposte; decision accuracy, response time, and valid-target accuracy can be recorded. In a variable-distance adaptive-lunge drill, the coach changes target distance before the cue, and the athlete adjusts footwork and lunge amplitude while maintaining technical control; valid touches and movement time can be monitored. These drills are hypothesis-informed examples, not interventions validated by the present study, and their dosage and effectiveness require prospective testing.

Motion-analysis technologies can provide objective descriptions of movement timing and technical execution (3, 22, 27). Recent fencing-specific studies describe lunge biomechanics, target-distance effects, and movement organization across fencing contexts (32–34). In practice, combining video analysis with coordination testing may support a broader assessment, but the present study does not establish the diagnostic validity of this approach or its ability to guide effective training. Any applied decisions should use standardized procedures and be evaluated in larger prospective studies. Biomechanics education nevertheless remains relevant to evidence-informed coach development (28).

### 4.5 Strengths and limitations

The present study has several strengths. To our knowledge, it is among the first to evaluate multiple coordination abilities alongside kinematic characteristics of the fencing lunge in youth foil fencers. Previous research has primarily described physiological or biomechanical characteristics of fencing performance (2–5, 22), whereas this study combined field-based coordination assessments with two-dimensional motion analysis. All participants completed the same defensive and offensive protocol under standardized conditions, supporting consistency in data collection. In addition, Spearman's rank-order correlation with Holm–Bonferroni adjustment provided a transparent and conservative approach to the 42 exploratory comparisons.

Several limitations should also be acknowledged. The census sample comprised only nine eligible competitive youth foil fencers, reflecting the limited availability of this population. The small sample size (*n* = 9) substantially limited statistical power, particularly for detecting moderate associations. Although the observed data included several large to very large correlation coefficients, the *post-hoc* power analysis indicated that the study was considerably less sensitive to moderate effect sizes. Therefore, nonsignificant findings cannot be interpreted as evidence of the absence of associations, and the observed relationships should be considered preliminary and hypothesis-generating pending confirmation in larger samples. Consequently, the findings should be regarded as exploratory rather than definitive. The cross-sectional design precludes causal inference. Two-dimensional Kinovea analysis cannot quantify three-dimensional joint kinematics, transverse-plane rotation, or complex multiplanar movement, although the software has shown acceptable validity and reliability for two-dimensional measurements (27). Recordings at 50 Hz provide a temporal resolution of 0.02 s per frame; this may introduce frame-level uncertainty when identifying the onset and completion of short actions and limits the assessment of rapid within-movement changes. The reported velocities should therefore be interpreted as average two-dimensional velocities rather than instantaneous peak velocities, and no acceleration measures were derived. Finally, the findings cannot be generalized directly to épée, sabre, adult fencers, or elite international competitors.

Future research should recruit larger multicentre samples across age groups, competitive standards, and fencing disciplines. Longitudinal and intervention studies are needed to determine whether targeted coordination training changes fencing biomechanics or performance. Higher-frame-rate three-dimensional motion capture, wearable inertial sensors, electromyography, and force-platform analysis may provide a more complete account of the underlying mechanisms. Future protocols should also prespecify primary associations, perform an *a priori* sample-size calculation, and limit the number of confirmatory comparisons. The present study should therefore be regarded as hypothesis-generating rather than confirmatory.

## 5 Conclusion

After adjustment for multiple comparisons, the present exploratory study did not identify any statistically significant associations between the assessed coordination abilities and fencing-lunge kinematics. The two largest coefficients, involving Reaction Ability with arm velocity during unsuccessful defensive actions and with front-leg velocity, were nominally significant before correction only; their adjusted *p*-values were 0.221 and 0.296, respectively. The results therefore do not provide confirmatory evidence that coordination abilities influence the biomechanical variables examined.

The observed coefficients should be viewed as candidate hypotheses for future adequately powered research. The study neither establishes causal mechanisms nor validates coordination tests as performance indicators for youth fencing. Larger prospective and experimental studies are required before specific training or assessment recommendations can be made.

The study's main contribution is a preliminary framework for integrating field-based coordination assessment with biomechanical observation in youth foil fencing. Its value lies in identifying testable questions and methodological priorities for future work rather than in providing definitive evidence of association.

## Practical applications

- Because no adjusted association was statistically significant, the present study does not support a specific training prescription or the use of coordination tests as validated performance indicators.
- A randomized choice-reaction parry–riposte drill may be evaluated: the coach delivers unpredictable attacks toward the septime or octave target area, and the athlete selects the appropriate parry before an immediate direct riposte. Defensive decision accuracy, response time, and valid-target accuracy can be recorded.
- A variable-distance adaptive-lunge drill may be evaluated: the coach varies target distance before the cue, and the athlete adjusts footwork and lunge amplitude while maintaining technical control. Valid touches, movement time, and movement quality can be monitored.
- These drills are hypothesis-informed examples rather than interventions validated by this cross-sectional study. Their training dosage, safety, and effectiveness should be established in prospective trials.

Future research should determine whether standardized coordination assessment and targeted training produce reproducible changes in fencing biomechanics and competitive performance.

## Data Availability

The raw data supporting the conclusions of this article will be made available by the authors, without undue reservation.
